# Advocating a rational use of self-confrontation micro-phenomenological interviews to improve performance in elite athletes

**DOI:** 10.3389/fspor.2024.1396197

**Published:** 2024-09-30

**Authors:** E. Terrien, M. Leblanc, J. Saury, D. Hauw, B. Huet

**Affiliations:** ^1^ Faculté des Sciences Sociales et Politiques, Institut des Sciences du Sport, Université de Lausanne, Lausanne, Switzerland; ^2^ Movement—Interactions—Performance (MIP), Nantes Université, Nantes, France

**Keywords:** self-confrontation interview, micro-phenomenological, enaction, performance, mental training

## Abstract

Self-Confrontation Micro-Phenomenological Interviews (SCMPIs) aim to capture the fine-grained details of an athlete's experience, focusing on pre-reflective consciousness without resorting to *post-hoc* rationalizations. This specific type of self-confrontation interview was developed in the domain of work analysis in cognitive ergonomics. Despite its integration into numerous research studies in sport performance and training practices, the direct effects of the SCMPI on athletes remain underexplored. In this perspective article, we provide a brief overview of the theoretical foundations of SCMPI as well as a description of the paradox of the SCMPI situation. Throughout this paper, we highlight the unsolved questions around use of SCMPIs to improve athletes’ performance by enhancing reflective thinking as well as pre-reflective awareness. Furthermore, we discuss the epistemological and practical considerations regarding the selection and use of activity traces in SCMPIs. The paper calls for further research to evaluate the SCMPI's impact on performance in elite athletes, emphasizing the need for a rational application of this method in both research and practical interventions.

## 1 Introduction

The self-confrontation micro-phenomenological interview (SCMPI) has been used in numerous research studies in sport (1–6). The SCMPI is a method consisting in confronting subjects with traces of their past activities (e.g., video recording of a training session), while asking specific questions designed to encourage the subjects to “re-live” their past experience and describe it following the chronological sequence of the events as they unfolded. The key aspect of these interviews is their aim to document and make a fine-grained study of an actor's experience during a past situation, while discouraging justification and retrospective rationalizations about this situation (7–10). Developed for research purposes, SCMPI situations are increasingly being integrated into training practices themselves (2, 11). Indeed, practitioners (e.g., coaches, sport psychologists) include the SCMPI in their interventions with a view to enhancing performance optimization in the short or medium term. However, to date, little is known about the effects of SCMPI situations on the participants. In most cases, the SCMPI is considered as a step in a process of documenting and analyzing an actor's experience (either for research or intervention purposes), but rarely as an intervention in its own right. In this perspective article, we begin by outlining the theoretical foundations of SCMPI. Then we describe what we call the SCMPI paradox, and we present it as a lever to improve reflective thinking as well as pre-reflective awareness, two pillars of athletes’ continuous improvement. Finally, we discuss the epistemological and practical considerations regarding the type and quality of traces of activity used in the SCMPI.

## 2 Theoretical foundations of the SCMPI

The SCMPI was theorized in the area of work analysis in cognitive ergonomics in the 1980s and implemented in numerous research studies referring to the course-of-experience framework (7, 9, 10, 12). The SCMPI method is inspired by von Cranach and colleagues’ self-confrontation interview methods (13), and Vermersch's (14) elicitation interview methods. The SCMPI uses the typical probes of Vermersch's elicitation interviews, while relying on traces of the actors’ activity to (a) “re-place actors at every instant in the past dynamical situation we are studying” [(8), p. 8]; and (b) to prevent the actor from reshaping their narrative in a fabulative manner (10). As stated by von Cranach & Kalbermatten [(15), p. 144]:

“In our studies, subjects were confronted with the video-film of their own action. An interview which evokes the actor's immediate report in such a situation is likely to reproduce with a fair quality what the actor can remember about his cognitive conscious experience during the act.”

During the SCMPI, the researcher guides the participant in a moment-to-moment chronological description of their intentions, expectations, sensations, actions, thoughts and interpretations related to the studied situation. Similarly to event-focused interviews (16), the primary goal of SCMPI use in sports and exercise activities is to generate insights into how people think, feel, and act. One specificity of the SCMPI is to make systematic use of recorded traces of the athletes’ past activity as in stimulated recall interviews [e.g., (17)]. SCMPI's other specificity is its grounding in an enactive theoretical framework of human cognition (i.e., the course-of-experience framework), making the assumptions that during the SCMPI the athlete does not merely recall the past situation, but re-enacts it while describing it through the expression of the pre-reflective consciousness. Indeed, the aim of the SCMPI is “to collect descriptions of the micro-dynamics of experience in its pre-reflective dimensions” [(8), p. 9]. The pre-reflective dimension of an actor's experience refers to Sartre's (18) conception of pre-reflective consciousness as an “immediate and non-cognitive form of self-awareness” [(8), p. 9]. Theureau (9, 10) defines the pre-reflective consciousness as the part of one's experience that an actor can show (e.g., by miming), relate and comment upon to an observer-interlocutor under favorable conditions. In practice, an SCMPI can take the form of the excerpt presented in Table 1.

**Table 1:** Excerpt of an SCMPI and description of the SCMPI process.

Excerpt of SCMPI	Description of the SCMPI process
Researcher: I let you comment on what you are doing, at this moment [referring to the unfolding action displayed on the screen], what you are trying to do, what you perceive, what you are doing, what you may be saying to yourself at this moment…	The researcher invites the sailor to describe his re-enacted experience. In this case, a video recording of a training session is used as the trace of the past activity. The researcher insists on “at this moment” as the goal is to document the sailor experience chronologically, moment to moment, as it happened during training
Sailor: well, start of the leg I think the wind is a little light, I see a puff coming in front of us so a little bit windward I'm trying to adapt the setting of my sail that is to tighten quite a lot the outhaul	The sailor starts describing his re-enacted activity
Researcher: yeah, quite a lot…?	The researcher encourages the sailor to continue and enrich the description, using an echo probe (19)
Sailor: well, practically flat out, but that is to say above all to have the feeling of having a sail that is fairly neutral, which carries me but which is fairly neutral in terms of stability, that is to say that allows me to open up if, to open up a little in phases where I'm going to try to luff up a little bit, or in any case to gain upwind uh, and that it's really uh, now there's a fairly well-centered downforce in the harness lines, so what I mention at one point and uh well for me it's a downforce which is important, that means that the foil and the equipment develop power and I manage to channel that and to retransmit that uh, a little in the opposite direction of the harness lines	The sailor enriches the description of the re-enacted activity by providing details on his expectations as the situation unfolds and the practical knowledge he mobilized during the action
Researcher: and so before, before doing this adjustment to re-stabilize your rig, do you anticipate the instability that there could be during the puff or do you already feel something at this moment?	The researcher seeks clarification about the chronology of the unfolding events to precisely document what is experienced by the rider at every moment, in terms of intentions, expectations, sensations, actions, thoughts, interpretations and emotions. Note that the phrasing of this prompt can be considered awkward from a theoretical standpoint, as it calls for a yes or no answer rather than aiming for the description of a re-enacted experience. However, it is sometimes useful for the researcher to use this type of prompt to move past uncertainty before continuing the interview. Furthermore, when asking “or do you already feel something at this moment”, the researcher invites the sailor to not limit his answer to yes or no but also to describe what did he felt at that moment
Sailor: no, I don't feel instability at this moment… well that was [the same as] yesterday's feeling it's that the puffs they were, it's very patchy on the water so, when you enter the puffs sometimes they are quite unstable so you don't know which way the wind was going to be when entering the puffs so my goal is above all to be very very stable and in some way solid when entering the puff so as not to be destabilized and to be very efficient right away, not to have a sail opening movement or I'll lose a few seconds of acceleration things like that so uh, that's why I try a lot of anticipate uh, on the settings and I don't wait to be in the puff to make the adjustment because I would have had to open my sail a little…	The sailor answers by clarifying his perceptions and expectations at this given moment. In this case, the sailor was familiar with SCMPI, but an additional probe by the researcher could have been necessary for athletes less familiar with this type of interview, especially after they had answered yes or no to the researcher's previous prompt

The excerpt is extracted from the research of Terrien et al. (6). The sailor is an elite rider of IQfoil (Olympic windsurfing class).

## 3 The SCMPI paradox

Interestingly, while the SCMPI aims at documenting an athlete's pre-reflective consciousness associated with a given sport situation, the SCMPI situation might seem paradoxical: when confronted to the traces of their past activity, the participant is put in a position to develop their situated reflection on their activity, but during the SCMPI, they are asked not to make use of this possibility (12). Indeed, it is known that confronting someone with traces of their past activity is likely to produce self-evaluation, and/or surprise or other emotions. Von Cranach and Kalbermatten (15) pointed out the need to use methods to “separate cognitions which actually happened during the episode from afterthoughts, and to locate cognition in the stream of events” [(15), p. 145]. During the SCMPI, the interviewer uses techniques to maintain the athlete in a state of “presence to past experience in the present situation” [(8), p. 10]. Guidance aims at bringing participants back to evocation “each time they shift from it towards the verbalization of generalities, comments, beliefs, judgments, explanations, or theoretical knowledge about it.” [(8), p. 11]. This is where the paradox of the SCMPI lies: the SCMPI situation affords the opportunity to the participants to analyze and reflect on past events as they are confronted with recorded traces of these events, yet they are asked not to make use of this possibility, but simply to express their pre-reflective consciousness at the moment of carrying out their activity (12).

In the next sections, we argue that SCMPI's potential benefits on an athlete's performance can be driven by two processes: enhanced reflectivity as well as improved pre-reflective awareness.

## 4 SCMPI to improve the athletes’ reflective thinking

During SCMPIs, researchers do their utmost to guide the participant in the re-enactment of the past situation so that the content of the participant's pre-reflective consciousness expressed during the SCMPI is similar to the content that would have been expressed during the original situation. As such, the SCMPI is one method among others to document an athlete's lived experience, and is assumed to be an efficient method to provide researchers with data to produce an acceptable symbolic description of the cognition of athletes (10, 20). It is obvious, however, that strictly speaking during an SCMPI the athlete does not relive the same situation as the one they experienced during their past experience. Indeed, as Høffding (21) pointed out, “what's done is done, the bullet's left the gun” (p. 133). In fact, the SCMPI documents the expression of the athlete's pre-reflective consciousness during the SCMPI itself and cannot give direct access to this pre-reflective consciousness during a past situation. However, we argue that it is both the analogy and the gap between the experienced situation and the “re-experienced situation” (during the SCMPI) that is likely to enhance an athlete's reflective thinking. That is, re-experiencing the past activity provides the athlete with a new opportunity for reflection-in-action (22), with the benefits of being in a safe environment and exempt of pressures of time or space, physical constraints, or motor control requirements inherent to the real sports situation. On the one hand, the gap between the original sports situation and the SCMPI situation is a limit to the documentation and analysis of the athlete's lived experience. On the other hand, it may benefit the participating athlete by allowing enhanced reflectivity.

Twenty years ago, Sève and Adé (23) presented the result of a case study in which they investigated the lived experience of a physical education teacher during an SCMPI. To do so, they conducted a first SCMPI with the teacher, based on the video recording of a physical education class, followed by a second SCMPI using the video recording of the first SCMPI. The second SCMPI revealed that the expression of the content of the teacher's pre-reflective consciousness during the first SCMPI was accompanied by the creation and reinforcement of knowledge about the past situation, surpassing the knowledge built during the physical education class situation. We argue that to date, this effect of the SCMPI on an athlete's reflective thinking has been both understudied and undervalued. Indeed, other authors have highlighted the role played by the SCMPI to improve performance in sport through the development of reflective practice (2, 11, 24–26). However in these studies, the SCMPI was included as an initial step of the intervention, prior to cooperative analysis, defining and testing solutions (11). Hauw (2) underlined the beneficial effects of SCMPIs for athletes as a means to rediscover the activity, to understand its organization and to consider new possibilities by formulating experiences and locating them physically and temporally. Moreover, when we use SCMPIs in research with elite athletes, they often express to us that they enjoy the SCMPI situation.

In the context of recent research with Olympic sailors, we conducted daily SCMPIs with a crew of Nacra 17 elite sailors during an international sailing regatta. SCMPIs were conducted after the training sessions 3 days before the regatta, and after each day of the regatta. In this work, the sailors were confronted with a set of data, which included video recordings with different camera angles as well as mechanical data (e.g., speed, angles of trim and heel). During the post-event debriefing, we asked the sailors about what they had learnt and how they had experienced the SCMPIs. Both crewmembers reported: “it's like doing two sailing sessions in one”. The helmsman highlighted how the SCMPI method catalyzes reflection:

“The fact of seeing ourselves again, hearing ourselves, saying, ‘Ah yes, why am I doing this, why am I doing that,’ yeah, that's really great. I totally agree that its like doing two navigations in one, it's tremendous, and especially you do it, but then you question yourself again, so you're putting your brain back into it a bit, and just by putting your brain into it for an hour, it's done, it's like you’ve gained a week's worth of experience.”

This excerpt further illustrates the paradox. Whilst the “why” questions are avoided by the interviewers (8, 9), the athletes do indeed engage in reflective thinking as they express their pre-reflective consciousness. However, to date there is a paucity of published empirical studies in the domain of sport performance optimization to support these effects reported by field researchers and practitioners.

## 5 Combining reflective thinking with the enhancement of pre-reflective awareness. The SCMPI as an efficient intervention to enhance continuous improvement in athletes

Together with reflective thinking about techniques and strategies, we argue that the SCMPI enhances pre-reflective awareness in the athletes. Indeed, during the SCMPI, the athlete is guided by the researcher to direct their attention to the *hic et nunc* (the present) of their past experiences (i.e., the re-lived situation watched on video) in order to describe their actions, sensations, emotions, and thoughts in a non-analytic, non-judgmental manner. In the context of a mindfulness and acceptance-based intervention with badminton players, Doron et al. (27) used self-confrontation interviews as a tool to practice mindfulness skills. They reported that self-confrontation interviews seemed to enhance the athletes’ awareness of their behavior and mental experiences in real performance situations. Unlike mindfulness training techniques that de-situate the athletes from real-life sport situations (e.g., sitting up straight with the instruction to focus on the breathing sensations (28), self-confrontation interviews maintain the athletes in a situation that is meaningful in relation to their sport. Furthermore, during the SCMPI's specific type of self-confrontation interview, the efforts made by the interviewers to keep the athletes focused on the present moment and express their pre-reflective consciousness, as well as the prompts typically used (e.g., at this moment… what are you doing? what are you thinking about? how do you feel?) may educate the athletes’ capacity to re-focus during training and competition. Future research should investigate to what extent the SCMPI can improve an athlete's mindfulness skills in comparison to other types of self-confrontation interview.

In the perspective of optimization of performance in elite athletes, reflective thinking together with the improvement of self-awareness combines two dimensions of continuous improvement (29, 30). Continuous improvement refers to the notion that instead of seeing their improvement leveling off despite increased practice, elite athletes continually improve their skills by gaining precise awareness of how the body feels, attentive awareness to movement, and mindful awareness, as well as by using reflective and metacognitive processes (29). Continuous improvement can be apprehended at various temporal scales (e.g., at the scale of a training session, a season, or several years). Future research should investigate the temporal scale at which the SCMPI has the greatest effect on continuous improvement in elite athletes.

## 6 What traces of activity to use and why?

The type and quality of traces used in SCMPIs impact the possibilities to re-situate the athletes in the past situation (31). That is, the traces used, and the way they are presented to the athlete, have an impact on the gap between the originally-experienced situation and the re-enacted experience during the SCMPI. The traces for SCMPIs are usually audio-video recordings [e.g., (5, 11, 24, 32)], but can also consist of other types of data such as the maps of a race (4, 33). Furthermore, artifacts can be used to help the participants to re-enact the situation and to mimic perceptive qualities and intensities that are difficult to express through verbalization, as was the case in the study by Leblanc et al. (32) in which a pair of reins was provided to the *écuyers* during the SCMPIs. However, there is a scarcity of information on the choices of the traces used by researchers or practitioners in SCMPIs. Indeed, in practice the choice of traces is guided by both epistemological and practical questions. The epistemological question is whether a trace used in an SCMPI provides contextual elements to better re-situate the athlete, without bringing anything new to the situation, or if this trace provides new elements that the athlete did not experience during the original situation. Indeed, confronting the athlete with traces of their activity or contextual elements that they are not immediately experiencing puts the athlete in an analytic position that is *a priori* incompatible with the aim of the SCMPI.

The practical questions are how to obtain the sufficient quality of traces given the constraints of the studied activity, and how these traces can be presented to the athletes during SCMPIs. Indeed, technological developments now allow researchers to collect audio-visual data in manners that are effective in both time and cost without having to transport bulky material, even in unfavorable environmental conditions such as in sailing [e.g., (34)]. However, to study the full length of a trail-running race lasting several days, choosing alternative traces such as maps of the race (4) can be considered a more practical option. Furthermore, the familiarity of the athletes with the chosen traces (e.g., a trace typically used for analysis during training) may facilitate the athletes’ involvement in the SCMPI situation. Future research should clarify the effects of different traces, contextual elements and interview settings on the athletes’ capacities to re-enact their past experience.

## 7 Conclusion: towards a rational use of SCMPIs in research and intervention with elite athletes

Throughout this perspective article, we have presented the SCMPI as a tool used by researchers and practitioners, and whose effects on performance in elite athletes remain to be rigorously evaluated. Indeed, we have argued that the paradoxical situation created by the SCMPI influences reflective thinking and self-awareness in the athletes. In this regard, the present paper is a call for a rational use of the SCMPI to improve the performance of elite athletes. Researchers and practitioners using the SCMPI should have a clear knowledge of the SCMPI's theoretical foundations and the possible implications of using this type of interview with the elite athlete population. Indeed, this type of interview should not be considered merely as a simple data collection, but as an intervention that can transform the way of thinking of the athlete who participates in it. Future research should assess how the participation of elite athletes in SCMPIs affects their performance by enhancing both pre-reflective awareness and reflectivity. Furthermore, future research should investigate how the type and quality of traces of activity selected as a support for SCMPIs influence the outcomes of these interviews.

## Data Availability

The raw data supporting the conclusions of this article will be made available by the authors, without undue reservation.
